# Supplementary material for: Design and research of automated warehouse simulation platform based on virtual visualization framework
Source: PeerJ Comput Sci. 2024 Jan 22;10:e1809. doi: 10.7717/peerj-cs.1809 (PMC11636691; doi:10.7717/peerj-cs.1809)
Supplement: Supplemental Information 1 [file peerj-cs-10-1809-s001.docx]

OSHWH

Interface name

****queryProductInventoryList4Page****

Request for clarification

| **Request content** | **Indications** |
| --- | --- |
| URL | <https://openapi.winit.com.cn/openapi/service> |
| Interface test | <https://sandboxopenapi.winit.com.cn/openapi/service> |
| Verification method | Token,md5 |
| Format | json |
| Character encoding | UTF-8 |
| The HTTP request mode | http |
| Request count limit | The default is 200 per minute, which can be increased if necessary. |
| Interface version | 1.0 |

Request in parameters

| **Name** | **Type** | **Required** | **Indication** | **Example** |
| --- | --- | --- | --- | --- |
| DOITier | String() | N | DOI layer： 1：30 below 2：30-60 3：60-90 4：90 above | 1 |
| inventoryType | String（10） | Y | Inventory type：Country：Country，Warehouse | Country |
| isActive | String（1） | N | Whether the goods are valid,Y/N | Y |
| pageNum | Integer（10） | Y | Page | 1 |
| pageSize | Integer（10） | Y | Display quantity per page | 100 |
| productCode | String（510） | N | Commodity Code | SKU1 |
| name | String（255） | N | Trade name | MM |
| warehouseId | String() | Y | Warehouse ID, click to query warehouse ID | 1000008 |
| warehouseCode | String() | N | Warehouse Code，Click to query the warehouse [Code](http://developer.winit.com.cn/tool/logistic.html) | DE0001 |
| startTime | String(20) | N | Inventory change start time  The start and end time span cannot exceed 24 hours | 2020-01-01 00:00:0 |
| endTime | String(20) | N | Inventory Change End Time  The start and end time span cannot exceed 24 hours | 2020-01-01 00:00:0 |

An example of a request

1. {
2. "action": "queryProductInventoryList4Page",
3. "app_key": "rebecca",
4. "client_id":"ODJKMDU1YZCTYJQ5YY00ZWZLLTK5N2QTOWY4MZI5OGMWNDG2",
5. "client_sign":"CC3F32A4D985B8176E22525F6ABD7FA1",
6. "data": {
7. "categoryID": "",
8. "DOITier": "",
9. "inventoryType": "Country",
10. "isActive":"Y",
11. "pageNum": "1",
12. "pageSize": "100",
13. "productCode": "",
14. "name": "",
15. "specification": "",
16. "warehouseId": "",
17. "warehouseCode": "DE0001"
18. },
19. "format": "json",
20. "language": "zh_CN",
21. "platform": "OWNERERP",
22. "sign": "8AB9BE71E5C0C9DBC5471327250B8A2C",
23. "sign_method": "md5",
24. "timestamp": "2015-06-16 00:19:26",
25. "version": "1.0"
26. }
